# Primary care during COVID-19 pandemic - a survey to establish clinical needs and lessons learned in infectious respiratory diseases in Spain

**DOI:** 10.1186/s12875-023-02160-z

**Published:** 2023-10-03

**Authors:** Manuel Linares, Laura Santos Larregola, Pilar Galicia García de Yébenes, Joaquín Santos Galilea

**Affiliations:** 1 Fundación iO, Calle Ortega y Gasset 60, 1D, Madrid, Spain; 2 Hospital Universitario Príncipe de Asturias, Madrid, Spain; 3 Centro de Salud Buenos Aires, Madrid, Spain

**Keywords:** Management, Primary care, Patient flow, COVID, Flu

## Abstract

**Background:**

The COVID-19 pandemic has exposed gaps and areas of need in health care systems. General practitioners (GPs) play a crucial role in the response to COVID-19 and other respiratory infectious diseases (e.g., influenza). Knowing the current flow of these patients and the real needs of GPs is necessary to implement new therapeutic and diagnostic strategies. We sought to learn about the flow of COVID-19 and flu patients in Spanish primary health centers and understand the training needs in both the diagnosis and treatment of these diseases.

**Methods:**

A total of 451 regionally representative GPs completed an online survey between January and February 2022.

**Results:**

Most of the GPs had available infection containment measures (79%) and access to point-of-care (POC) rapid diagnostic testing (81%) in their centers. The availability of on-the-day diagnostic tests for COVID-19 was higher than that for influenza (80% vs. 20%). Most GPs referred 1 of 10 COVID-19 or flu patients with moderate to severe disease to the emergency department (80% and 90%, respectively). Training/knowledge was considered good regarding diagnostic tests and vaccines (85%) but null or low regarding antivirals (60%) and monoclonal antibodies (80%).

**Conclusions:**

This survey identified the conditions of Spanish GPs in terms of the diagnosis and treatment of COVID-19 and flu patients. Respondents’ comments suggested that quite radical system-level adjustments are needed to allow GPs to capitalize on the potential benefits of POC tests for diagnosis, reduction of referrals, and monitoring of these diseases.

## Background

Primary care is the first line of defense for the health care system during a pandemic. General practitioners (GPs) work with other health care professionals, allowing cohesion in the health care system and protecting patients and communities [1].

The COVID-19 pandemic has exposed gaps and areas of need in health systems worldwide [2]. According to the World Health Organization (WHO), primary care plays a major role in the COVID-19 response [3]. GPs play a crucial role in the struggle against the pandemic as the first point of contact for possibly infected patients and are responsible for the short- and long-term follow-up care of most COVID-19 patients [1]. In addition to their role in monitoring COVID-19 patients, GPs have a relevant function in the screening and monitoring of patients with other infectious respiratory diseases, such as influenza. Moreover, despite COVID-19 vaccination, simultaneous outbreaks of COVID-19 and flu might overwhelm health systems [4]. By performing early diagnoses and differentiating patients with respiratory symptoms from those with COVID-19 or influenza, GPs reduce the demand for hospital services and the economic burden in health care. The arrival of the influenza season increases the need for prevention, diagnosis, management, and capacity across health care centers. Influenza leads to substantial direct and indirect health care burdens, which vary depending on the health status of the infected individual and the treatment setting [5]. A careful approach is needed to prevent health services from being overwhelmed by a surge in demand that could exceed capacity. Point-of-care (POC) testing presents new possibilities for the management of influenza patients presenting to health care providers with acute respiratory symptoms [6]. Following Schols et al., the international definition of POC testing is testing to support clinical decision-making that is performed by a qualified member of the practice staff near the patient and on any part of the patient’s body or its derivatives during or very close to the time of consultation to help the patient and physician decide upon the most suitable approach; the testing results should be known at the time of clinical decision-making [7]. Accuracy rates of POC tests are approximately 80% for identifying COVID-19 infections [8]. Hence, improving POC testing, including both molecular and rapid antigen testing, can potentially support GPs in quickly identifying infectious and noninfectious respiratory diseases (e.g., COVID-19 and flu), inform patient triaging, and improve clinical management [9].

Although the role of primary care and GPs in health emergencies such as COVID-19 and flu is well known, different studies have revealed gaps involving primary care in pandemic preparedness and response planning [10]. Kierkegaard et al. performed a survey among GPs in England that identified that GPs’ knowledge of POC testing influences their degree of trust, uncertainty, and perception of the risk of POC testing. Moreover, in this study, GPs expressed concerns about funding, occupational exposure, and workload in providing POC testing [9].

To successfully manage the current COVID-19 crisis and new future pandemics, we need to understand the impact and challenges faced by GPs in Spain. In this study, we aimed to investigate the challenges encountered and solutions implemented by GPs when they faced SARS-CoV-2 and influenza patients in Spanish primary health care centers and to understand the training needs from both diagnosis and treatment perspectives.

## Methods

This paper reports on data from an online survey. GPs answered questions on diagnostic tools, management of suspected cases of SARS-CoV-2 and influenza and training in new treatment and diagnostic strategies. Scientific endorsement was obtained from all the scientific societies that participated in this study [Sociedad Española de Médicos de Atención Primaria (SEMERGEN), Sociedad Española de Medicina de Familia y Comunitaria (SEMFYC) y Sociedad Española de Médicos Generales y de Familia (SEMG)]. This study was conducted in accordance with the Declaration of Helsinki to guarantee the anonymity of all participants.

### Participant selection and recruitment

All GP members of Fundación iO and 3 Spanish societies (SEMERGEN, SEMFYC and SEMG) were invited to participate in the online survey in February 2022 through the communication channels of each society (newsletter, email, etc.). Reminders were sent out in March and April 2022. The survey was closed on May 13, 2022. The URL leading to the online survey was sent to the participants with all the following information: the aim and importance of this study, the voluntary nature of participation, data security and anonymity of the survey. Survey completion was interpreted as informed consent to the anonymous use of the data. Participants did not receive incentives or funding to participate.

### Survey questions

The online survey had 19 closed-ended questions without open fields or adverse effect questions. The survey was on the diagnostic tools, patient referrals, patient flow at the primary care centers in Spain and training needs. The survey questions are provided in Table 1. The questions were written by Linares M with feedback and validation from 25 primary care experts. Based on the comments from the experts, Linares M wrote the final questions.

**Table 1:** Wording of closed-ended questions in the online survey

Closed-ended survey questions	
1	Autonomous community of Spain (Location of practice)
2	Indicate your age
3	Indicate your sex
4	Do you have a specific flow organized for patients who come to your center with respiratory symptoms (“infection containment measures “)? (Yes/No)
5	Do you have point-of-care (POC)/rapid response testing at your facility? (Yes/No)
6	Regarding the diagnostic capacity in your workplace, at this time, you have… (Yes/No)
	a) SARS-CoV-2 PCR tests
	b) SARS-CoV-2 antigen tests
	c) SARS-CoV-2 serology tests
	d) Influenza diagnostic test (PCR or rapid test)
	e) Respiratory syncytial virus, RSV test (PCR or rapid test)
	f) Chest radiography
	g) C-reactive protein level assessment
7	How long does it take to get the results? (minutes/on the same day/48 to 72 h)
	a) SARS-CoV-2
	b) Influenza
	c) RSV
8	Do you think you have enough information about diagnostic tests for COVID-19 and flu? (Yes/No)
9	What percentage of your patients come to their consultation having already self-tested? (< 5%/5 to 20%/20 to 50%/> 50%)
10	What percentage of patients are referred to the hospital for moderate or severe COVID-19? (< 10%/10–25%/25–50%/> 50%)
11	What percentage of patients are referred to the hospital for moderate or severe influenza? (< 10%/10–25%/25–50%/> 50%)
12	When referring a patient with COVID-19 or flu to the emergency room, which of these criteria do you consider most important in your usual clinical practice (presence of symptoms, stable referral criteria, presence of comorbidities, need to be seen by another specialist)?
13	How would you define your relationship with hospital care/emergency providers (no relationship/poor/normal/good/excellent)?
14	Do you think that treatment with antivirals prescribed during the primary consultation for COVID-19 would be effective? (Yes/No)
15	Do you think that treatment with antivirals prescribed during the primary consultation for flu would be effective? (Yes/No)
16	Do you think it is possible that in the future primary care providers will prescribe antiviral treatments for COVID-19 and flu patients? (Yes/No)
17	Define your level of training/self-knowledge in (none/low/medium/high)/:
	a) Diagnostic tests
	b) Antivirals
	c) Monoclonal antibodies
	d) Vaccines
18	Which of the following criteria would you consider helpful in your work (more diagnostic resources, improve relationships with other health professionals, improve my training/patient’s follow-up/therapeutic resources)?
19	From 1 to 10, how would you define your training in handling the pandemic?

### Data analysis

No formal sample size was calculated. The sample size was defined as the total number of GPs who responded to the questionnaire. A descriptive analysis was performed. Medians and percentages were calculated for continuous and categorical variables, respectively. The data were managed with SPSS v20.0 (IBM Corp., Armonk, NY, USA).

## Results

### Sample characteristics

A total of 451 regionally representative GPs from 15 autonomous communities completed the online survey (1.67% response rate). The autonomous communities that were more represented as locations of practice were Andalucía and Madrid, with 253 and 54 questionnaires completed, respectively. The sociodemographic characteristics of the sample are displayed in Table 2. Participants comprised mostly women (55%) and had an average age of 52 years.

**Table 2:** Sample characteristics

	*n*	*%*
*Age*		
30–49	162	37
50–65	253	58
≥66	20	5
*Sex*		
Female	246	55
Male	200	45

### Infection containment measures and point of care (POC)

Most of the GPs (79%) reported having infection containment measures at their primary care unit to respond to patients with respiratory symptoms (defined as a group of measures that prevent and contain the spread of infectious diseases in the centers for the management of patients with respiratory symptoms, including hand hygiene, personal protective equipment, contact and airborne precautions, etc.). Moreover, 81% of the GPs confirmed fast access to POC diagnostic tests. Almost 20% of the GPs noted that more than half of the patients who attended primary care centers had undergone a COVID-19 diagnostic self-test prior to the consultation.

### Diagnostic capacity

**Fig. 1:**
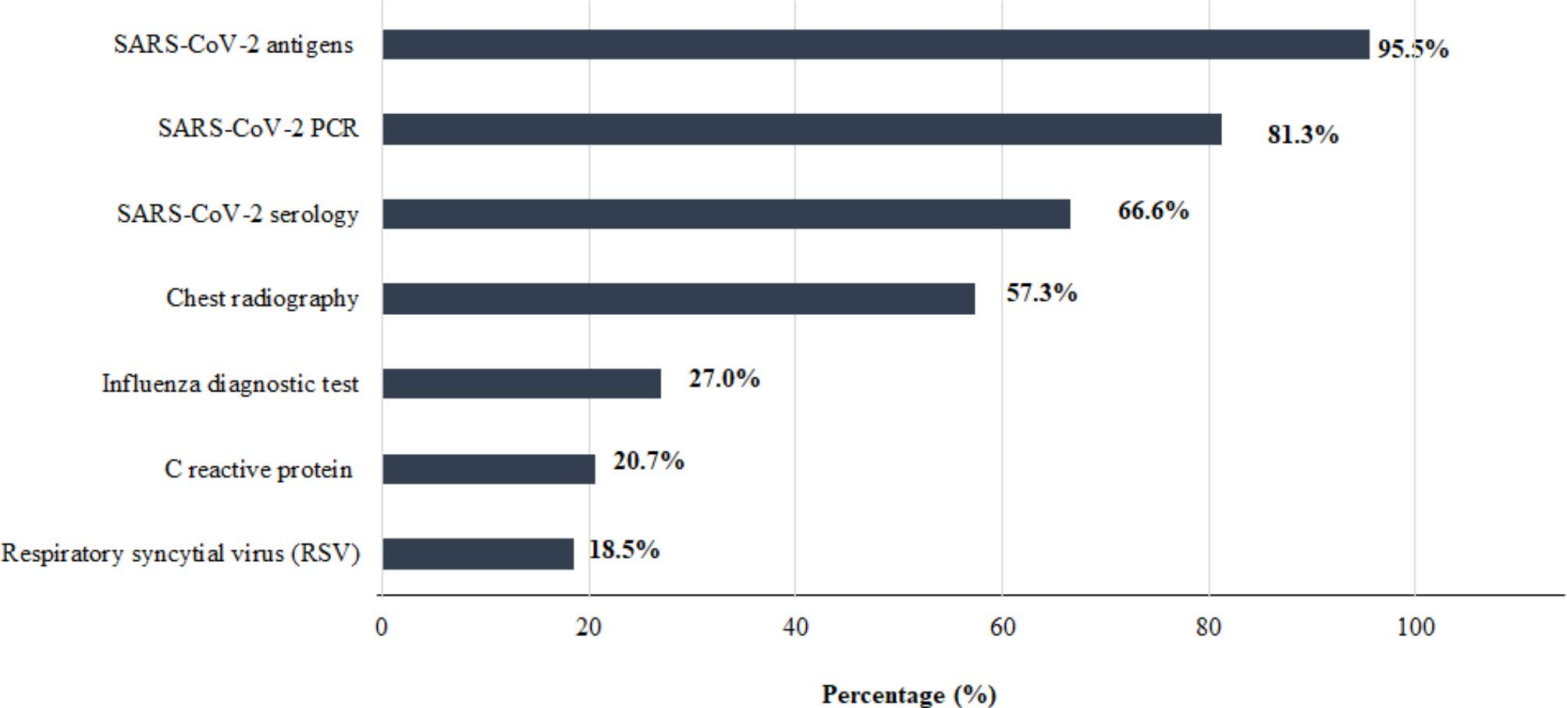
Diagnosis capacity of GPs at Spanish primary center care

Most of the GPs (95%) had antigen COVID-19 diagnostic tests available, followed by PCR (81%) and serology (66%). On the other hand, only 27% and 18.5% of GPs had access to influenza and respiratory syncytial virus (RSV) diagnostic tests, respectively. All the data related to access to diagnostic tests are described in Fig. 1.

### Time to diagnosis

**Fig. 2:**
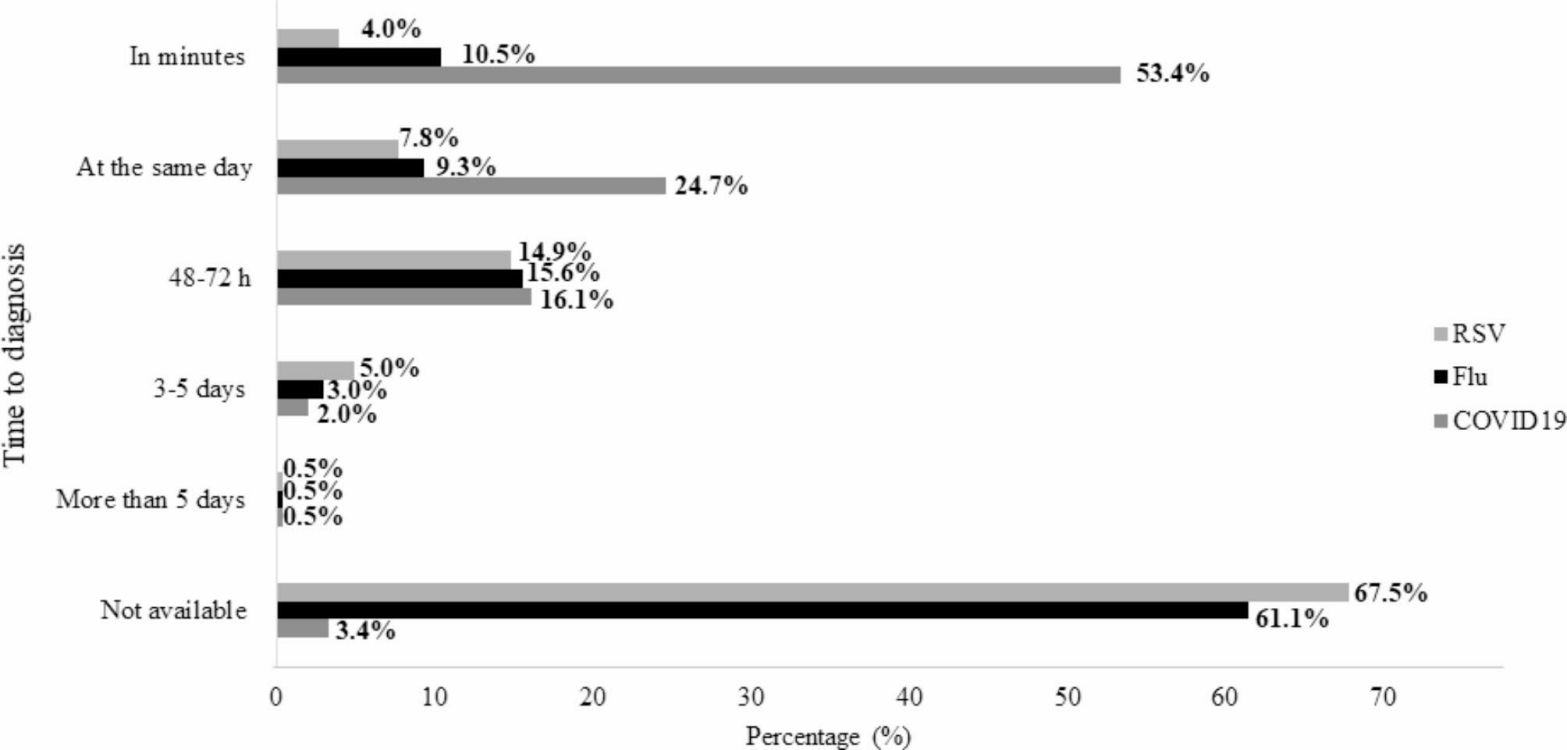
Time for diagnosis by GP from COVID-19, Flu or RSV

Most of the GPs (53.4%) could diagnose COVID-19 within minutes. Only a few GPs had access to a diagnostic test for flu or respiratory syncytial virus (RSV) and could make a diagnosis of the infection on the same day (20 and 11%, respectively). Data on time to diagnosis are presented in Fig. 2.

### Patient referral

More than 80% of GPs referred 1 of 10 patients diagnosed with COVID-19 who had a risk of moderate to severe disease (with comorbidities) to emergency departments. A similar scenario was observed for the flu, with 90% of GPs referring patients. The principal cause of referring a patient with COVID-19 or flu to the emergency room was the presence of symptomatology (60.8%), followed by stable referral criteria (20%), presence of comorbidities (18.5%) and finally the need to be seen by another specialist (0.8%).

### Emergency and hospital care relationships

Only 21% of primary care doctors declared that they had a good or excellent relationship with emergency or hospital care. Most of the GPs considered the relationships with these health care professionals normal (58%) or poor (12%). For 9% of GPs, this contact was nonexistent.

### Antiviral treatment

In the opinion of 65% of the GPs, antiviral prescriptions against COVID-19 are an effective treatment for patients at the primary care evaluation. A similar scenario was observed for flu (57%). Most of the GPs (77%) considered that in the future, it will be possible to prescribe antivirals to COVID-19- and flu-positive patients in primary care.

### GP training/self-knowledge

**Fig. 3:**
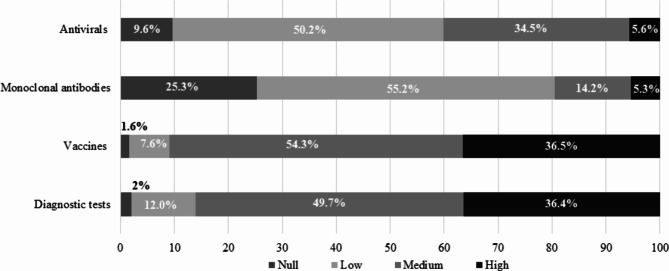
GP training and self-knowledge

**Fig. 4:**
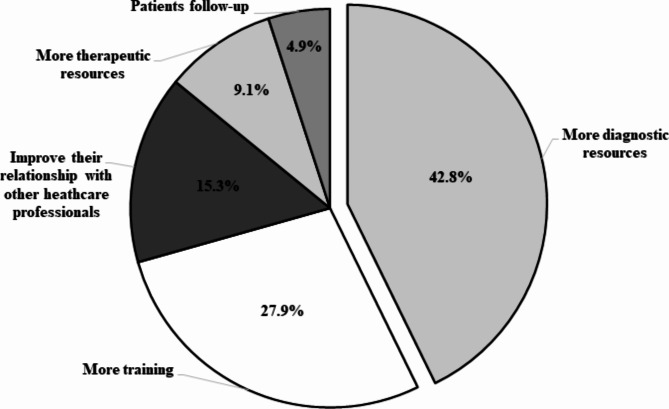
Criteria to improve primary care

More than 85% of GPs reported that they knew about diagnostic tests or vaccines (Fig. 4). However, 60% and 80% of GPs stated that they lack *self-knowledge* of antivirals or monoclonal antibodies, respectively (Fig. 3). Most of the GPs considered that their COVID-19 management was satisfactory (7.28 on a 10-point scale).

### Criteria for improvement of primary care

A great proportion of GPs (43%) stated that having more diagnostic resources at primary care centers will help them perform their job. Having more experience (by getting trained on diagnostic techniques such as diagnostic tests, antivirals, monoclonal antibodies and vaccines) and improving their relationship with other HCPs can help their performance, according to 28% and 15% of the surveyed GPs, respectively (Fig. 4).

## Discussion

This study describes the current situation in primary care regarding the diagnosis and treatment of respiratory diseases through the perspective of 451 GPs from 15 autonomous communities (regions) in Spain.

GPs are placed at the center of the health care system since they have a vital role in emergencies [1]. They can easily assess and manage infectious diseases such as seasonal influenza [11] and have widely recognized involvement in pandemic preparedness [12]. Indeed, this study showed that less than 10% of patients diagnosed either with COVID-19 or flu with moderate to severe risk (with comorbidities) were referred to hospitals by most GPs. This is in accordance with other studies that showed that access to a GP is associated with lower emergency department use [13]. In contrast, patients without a GP are more likely to go to the emergency department and have a higher proportion of nonurgent consultations [13].

Even so, GPs experience many challenges in fulfilling their role [1]. Particularly, as the pandemic decelerates, COVID-19-related complications must be addressed alongside robust control of influenza infections to avoid outbreaks that can lead to increased bed occupancy in hospitals and considerable financial implications [14]. Therefore, better approaches to sustain primary care services need to be considered.

This survey showed that not all GPs had infection containment measures at their primary care center (79%) to treat patients with respiratory symptoms. Unfortunately, this number may be lower as the pandemic ends and media interest in the prevention of respiratory diseases decreases. Most of the GPs (95%) had access to COVID-19 antigen tests, 81% to PCR, and 66% to COVID-19 serology tests. A possible explanation for these results is that POC antigen tests are authorized by the WHO and are particularly useful in case PCR results are not available in a timely manner or in case of an overwhelmed health care system [15], which can happen in Spain. A total of 27% of GPs responded that they had access to influenza diagnostic tests, and only 18.3% had access to diagnostic tests against RSV. For influenza, accurate and rapid molecular test platforms have become available in recent years, with a substantial impact on reducing nosocomial transmission, reducing the use of routine blood tests, blood cultures, and chest radiography by 20%, 18%, and 19%, respectively, and improving patient flow by adopting better infection control measures [16–18]. However, the survey demonstrated that the clinical diagnosis of viruses with public health impacts, such as influenza and RSV, continues to present a challenge for most physicians in Spain and needs to be better integrated into primary care [19, 20]. In response to this, sentinel syndromic surveillance systems will be set up in Spain for influenza, RSV, and SARS-CoV-2; this will involve the provision of new testing pathways, the use of PCR tests rather than antigenic tests, the reestablishment of regional networks, and the establishments of new networks. Finally, only 57% of GPs responded that they had access to chest radiography, and an even smaller percentage responded that they had access to CRP tests (Fig. 1), which can have critical implications. Chest radiography is an important diagnostic method for evaluation of the airways, pulmonary parenchyma and vessels, mediastinum, heart, pleura and chest wall that can help to ameliorate patient management, as shown by Speets et al. [21]. On the other hand, CRP levels that are positively correlated with the severity of different infections have been recently described as a good predictor of COVID-19 severity [22]. Not only can CRP levels predict COVID-19-associated severe pneumonia early [23], but they were also shown to be elevated in patients with a poor COVID-19 prognosis, in patients who dies from COVID-19-related causes, and in patients with additional COVID-19-related radiological lesions and tissue damage [22, 23].

POC testing enables GPs to make more trustworthy decisions and to perform quicker interventions, increasing efficiency (e.g., improving the quality of antibiotic prescriptions and reducing the number of prescriptions) and consequently benefiting morbidity and mortality rates [7, 24]. Although 81% of the GPs reported that they had instant access to POC tests, the above results showed a lack of access to some of the most important tests (Fig. 1), which can indicate that the POC concept is not very well understood among GPs. This may be due to the rapid emergence and evolution of POC tests in the last few years, particularly following the COVID-19 pandemic [25].

The survey further demonstrated a high frequency of rapid COVID-19 self-tests performed by patients prior to consultation, highlighting their high availability at local pharmacies as a way to support Spanish primary care. However, the timing from diagnosis to results can still be an issue, as 2% of GPs reported a period of 2 to 5 days to have the results for a COVID-19 diagnosis (Fig. 2). A worse scenario was observed for influenza and RSV detection, with only 20 and 11% of the GPs having the diagnostic results on the same day, respectively.

Kierkegaard et al. showed that GPs’ knowledge of POC tests influences their degree of confidence in POC test use [9]. Here, 85% of the participants were considered to have satisfactory knowledge about vaccines and diagnostic tests (Fig. 3). The results showed that 65% and 57% of the GPs considered that antiviral prescriptions against COVID-19 and flu, respectively, were an effective treatment for patients in primary care. In contrast, more than 50% of GPs reported having poor knowledge of antivirals and monoclonal antibodies against SARS-CoV-2 or influenza infection. These treatments given at an early stage of the infection can substantially reduce the risk of hospitalization and death among high-risk patients [26]. Therefore, facilitating better training in diagnostic techniques for Spanish GPs could be an effective approach to improving the treatment of patients with respiratory infections in primary care. In addition, further analysis should be performed to underpin major barriers to more efficient testing and develop future guidelines, such as the reinforcement of POC testing in highly populated regions to decentralize testing from hospitals, appropriate training for all health care providers, not only GPs, and the establishment of testing regulations (e.g., POC testing in combination with previous self-testing only for high-risk patients, elderly individuals, and health care professionals). Moreover, an additional measure to reduce the economic burden may rely on investing in diagnostic tests that can detect various infections all at once (e.g., the FilmArray® Respiratory Panel) [27].

The postpandemic health system in Spain faces an enormous challenge in reforming the organization of the national health system to respond to patients’ health needs. However, this can only be achieved by strengthening primary health care and improving the relationship with hospitals/emergency rooms to guarantee adequate conditions for GP performance [28]. In this survey, only 21% of primary care doctors declared that they had an excellent/good relationship with emergency or hospital care providers. To improve this interaction, the Spanish government recently announced the creation of a Public Health Agency. By preparing efficient plans for alerts, risks, and current or emerging threats, this governmental agency will perform crucial roles such as public health surveillance, preparation and response to future emergencies, advice and evaluation assessment of international public health policies, and health risk communication to better respond to societal health care needs and possibly reinforce POC testing strategies [29]. Moreover, the Spanish government recently approved 172 million euros to finance the Action Plan for Primary and Community Care 2022–2023. The investment will certainly increase the resolution capacity in primary care and allow the acquisition of equipment, as well as optimize administrative processes and promote the quality of care [30].

Although the number of respondents was high (451 GPs, the largest number in a study on primary care in Spain), this study presents some limitations. The present study examined a convenient sample from a limited list of all registered general practitioners in IO foundations and 3 Spanish societies. However, the sample represented solely 1% of the GPs in Spain [31]. Additionally, the level of knowledge of each GP about tests and treatments for respiratory diseases was underexplored and may not have represented the overall knowledge of the GP population. Additionally, the survey questions were highly subjective, and future efforts should be considered to adopt a more objective questionnaire (e.g., years of experience with diagnostic techniques; how many tests performed per day). Another caveat of this study is that in the timeline during which the questionnaire was open (2 months), several aspects related to the COVID-19 pandemic situation changed (e.g., changes in the number of positive COVID-19 patients), which could have introduced some bias to the results. Finally, in terms of the percentage of patients referred to hospitals, there was no information regarding the disposition rate from the emergency department. Future studies should include the number of patients referred together with the number of patients admitted vs. sent home.

## Conclusions

After the major role of primary care was emphasized during the COVID-19 pandemic, it is necessary to have a strategic plan that allows investment in primary care infrastructure and needs. Infection containment measures at primary care centers, the availability of diagnostic resources for respiratory diseases and the overall level of expertise on new treatments should be increased to improve GPs’ management of patients with respiratory symptomatology. Moreover, it is critical to take into account GPs’ knowledge and opinions about diagnostic and treatment strategies for SARS-CoV-2 and influenza to enable appropriate implementation strategies. Future research should explore appropriate contexts for POC test use and implementation in primary care.

## Data Availability

All data generated or analyzed during this study are included in this published article.
